# Mut2Vec: distributed representation of cancerous mutations

**DOI:** 10.1186/s12920-018-0349-7

**Published:** 2018-04-20

**Authors:** Sunkyu Kim, Heewon Lee, Keonwoo Kim, Jaewoo Kang

**Affiliations:** 1 Department of Computer Science and Engineering, Korea University, Seoul, Korea; 2 Interdisciplinary Graduate Program in Bioinformatics, Korea University, Seoul, Korea

**Keywords:** Mut2Vec, Distributed representation, Deep learning, Mutation embedding, Cancer

## Abstract

**Background:**

Embedding techniques for converting high-dimensional sparse data into low-dimensional distributed representations have been gaining popularity in various fields of research. In deep learning models, embedding is commonly used and proven to be more effective than naive binary representation. However, yet no attempt has been made to embed highly sparse mutation profiles into densely distributed representations. Since binary representation does not capture biological context, its use is limited in many applications such as discovering novel driver mutations. Additionally, training distributed representations of mutations is challenging due to a relatively small amount of available biological data compared with the large amount of text corpus data in text mining fields.

**Methods:**

We introduce Mut2Vec, a novel computational pipeline that can be used to create a distributed representation of cancerous mutations. Mut2Vec is trained on cancer profiles using Skip-Gram since cancer can be characterized by a series of co-occurring mutations. We also augmented our pipeline with existing information in the biomedical literature and protein-protein interaction networks to compensate for the data insufficiency.

**Results:**

To evaluate our models, we conducted two experiments that involved the following tasks: a) visualizing driver and passenger mutations, b) identifying novel driver mutations using a clustering method. Our visualization showed a clear distinction between passenger mutations and driver mutations. We also found driver mutation candidates and proved that these were true driver mutations based on our literature survey. The pre-trained mutation vectors and the candidate driver mutations are publicly available at http://infos.korea.ac.kr/mut2vec.

**Conclusions:**

We introduce Mut2Vec that can be utilized to generate distributed representations of mutations and experimentally validate the efficacy of the generated mutation representations. Mut2Vec can be used in various deep learning applications such as cancer classification and drug sensitivity prediction.

**Electronic supplementary material:**

The online version of this article (10.1186/s12920-018-0349-7) contains supplementary material, which is available to authorized users.

## Background

Mutation representation by simple binary values (e.g., each existing mutation is given a value of 1; if a mutation does not exist, it is given a value of zero) has been commonly used in various machine learning models designed for cancer analysis. However, since binary representation does not capture mutational context (e.g., mutations that frequently co-occur, distinction between driver mutations and passenger mutations), it provides insufficient information for cancer analysis such as cancer subtype classification, patient clustering, or drug sensitivity prediction. Although significant amounts of mutations have been discovered due to advances in sequencing techniques, it is generally known that passenger mutations have no role in cancer progression. In contrast, driver mutations directly affect cancer progression, and they tend to be observed frequently in the cancer profiles of patients. Applying these important mutational properties to mutation representation is critical for improving cancer analysis. Furthermore, if a mutation representation captures the characteristics of driver mutations, it is possible to discover novel driver mutations by calculating the similarity between a candidate mutation and each of the driver mutations. Based on this motivation, we aim to address the problem by developing continuous and distributed representations of mutations using deep learning techniques.

Recently, Deep Learning, one of the artificial neural network-based machine learning techniques has been making remarkable improvements in various applications such as text mining [1], speech recognition [2], image classification [3] and even the prediction tasks in biomedical domain such as protein secondary structure prediction [4] and DNA-protein binding prediction [5]. Various continuous distributed representations were introduced to be jointly used with deep learning models. Word2Vec [6] is one of the well-known models trained to represent words in continuous space. This model is a multi-layered neural network consisting of an input layer, embedding lookup layer, and prediction layer. For the representation of documents in a continuous space, Doc2Vec [7] which is an extension of Word2Vec, adds document vectors to the embedding lookup layer. Since the distributed representation of words includes semantic relationships among vocabularies such as the semantic similarity between two words, the representations can contain additional information compared with binary representation which contains information on the existence of words.

Similar attempts to represent data in a continuous vector space have been made in the biomedical domain. ProtVec [8] applies Word2Vec to a protein sequence to obtain distributed representations of a 3-gram amino acid sequence. The protein sequence is initially split into 3-grams each having a biological significance and regarded as a “word”. The next step is to run the Word2Vec algorithm using Skip-Gram. Seq2Vec [9], which extends the approach of ProtVec, applies Doc2Vec to represent a sequence not just by combining all the sequential elements of ProtVec 3-grams, but by directly embedding the sequence itself. Finally, Dna2Vec [10] generalizes the 3-gram structure of ProtVec and Seq2Vec to a k-gram structure. Another approach involves SNP2Vec [11], which embeds individual SNPs into a continuous space by using a denoising autoencoder [12] and Diet Networks.

Nevertheless, since Skip-Gram relies on co-occurrence information between data units (words or k-grams), it is difficult to guarantee the quality of the vectors if the input data lacks co-occurrence information. To address this issue, some studies that apply existing structured or graph knowledge to embedding processes have been introduced. RC-NET [13] adds two regularization functions to the Skip-Gram objective function, which capture the relational distance between the words based on their categorical information. Faruqui et al. [14] proposed a method that applies synonym-based graph knowledge to existing word vectors. Using a simple mathematical process, graph information is added to the word vector while information on its previous state is preserved.

In this work, we propose a novel pipeline, Mut2Vec, to generate distributed representations of mutations for the characterization of cancer cells. Because our vector space captures the characteristics of driver mutations and distinguishes driver mutations from passenger mutations, it has the potential to improve performance in other applications. Our mutation vectors can help identify driver mutations by investigating the vector space. We hypothesized that when an unidentified mutation is near many driver mutations in the vector space, the mutation could be a candidate driver mutation. Our mutation vectors can also help machine learning applications capture important biological information and yield better results than conventional binary representation. We assume that mutations are critical to the development of cancer when they co-occur in many cancer samples. Our assumption is similar to the text mining assumption that words are semantically meaningful when the words co-occur in many sentences. Word embedding algorithms such as Skip-Gram utilize co-occurrence information to embed words in a semantically meaningful distributed continuous space that places words with similar meanings close to each other. In this work, we attempt to leverage such word embedding techniques to embed gene-level mutations in a continuous distributed space that captures the semantic relations among the cancerous gene-level mutations.

To produce precise mutation vectors, a sufficient amount of information on co-occurring mutations is needed. However, the number of cancer samples with co-occurring mutations is limited. In the case of the Google News corpus, which is a standard text corpus for training word vectors, there are more than 100 billion tokens for three million words. In comparison, the database of the International Cancer Genome Consortium (ICGC) [15] has only about 13,000 cancer samples for more than 20,000 mutated genes. Because of this limitation, it is difficult to make reliable observations of co-occurring mutations, which is essential to producing high quality embedding. As a result, rare mutations do not have enough information on co-occurring mutations, so they do not learn proper mutation vectors. Therefore, these mutation vectors are placed in the wrong location on the vector space and act as noise in the analysis using distance between vectors such as clustering. To resolve this problem, we utilized biomedical literature and a protein-protein interaction (PPI) network to enhance the quality of mutation vectors.

To evaluate our embedding process, we visualized driver mutations and passenger mutations using our vectors. We confirmed that the two mutation groups were mutually exclusive to each other. The experimental results demonstrate that our mutation vector can determine whether each mutation is a driver or passenger mutation. We also identified driver mutation candidates using a clustering method. To evaluate the candidates and confirm their validity, we referenced recent biomedical literature in which true driver mutations are reported.

## Method

Cancer cells do not arise from random combinations of mutations. Cancer cells are due to their accumulated mutations that occurred during their evolutionary process [16]. Though mutations are abnormal in terms of their origin, their occurrence is inevitable. From this aspect, we set these co-occurring gene mutations in a sample as “context.” Among them, we also exclusively selected protein-altering mutations. Using the Skip-Gram model, we constructed the basic Mut2Vec model and obtained basic Mut2Vec vectors, where each vector is a 300-dimensional distributed representation of mutations and contains co-occurrence information of gene mutations from ICGC dataset.

However, there still exists an insufficient amount of data in the biomedical domain, compared with other domains such as the Natural Language Processing (NLP) domain. In the biomedical literature, gene names are mentioned in their biological context. By extracting contexts from the literature and adding them to our vectors, we overcome the limitations of data insufficiency and enhance the vectors to capture more precise gene-level mutational properties. We used the Skip-Gram model to train word representations on PubMed abstracts. Based on the learned word representations, we initialized the weight matrix of the embedding lookup layer with the word vectors of each gene when training mutation representations on the ICGC dataset. Our Mut2Vec+PI (PubMed Initialized) model initializes mutation vectors using PubMed word vectors and trains the Skip-Gram model on the ICGC dataset using the initialized vectors. Furthermore, we added structured biological knowledge using the PPI network BioGRID [17]. Assuming similar proteins are involved in similar cellular processes and their alteration effects are alike, we utilized a retrofitting process to post-process the output vectors [14]. Our Mut2Vec+R (Retrofitted) model applies retrofitting to the basic Mut2Vec output. Our Mut2Vec+PI+R model employs both PubMed initialization and retrofitting.

**Fig. 1:**
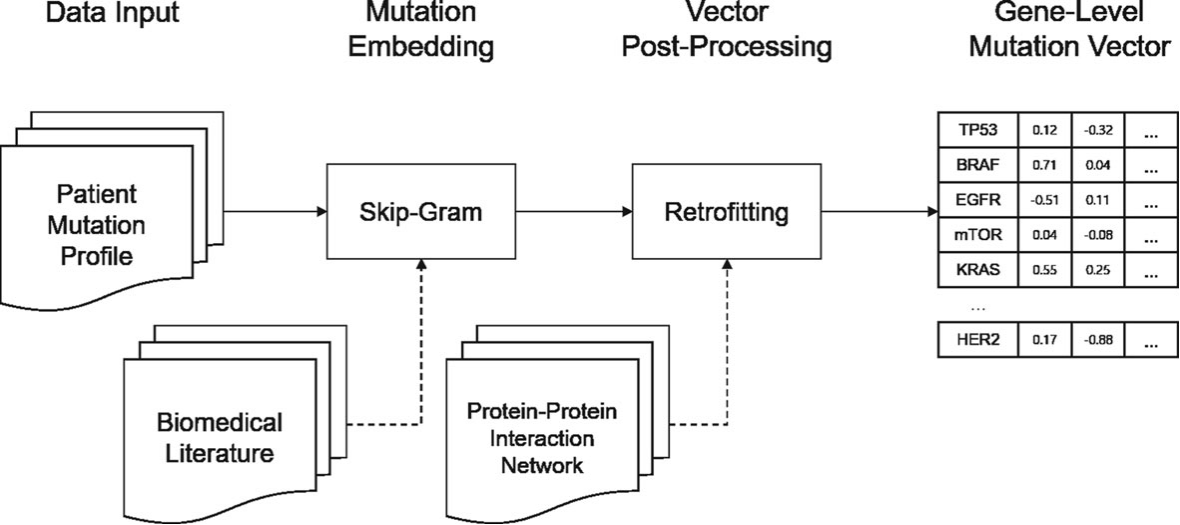
The overview of Mut2Vec Pipeline. Our pipeline is composed of two modules: an embedding module based on Skip-Gram and a vector post-processing module equipped with retrofitting. In our pipeline, we make four mutation embedding models. The first model uses only the Skip-Gram module on mutation profiles, and we call the model basic Mut2Vec. In our Mut2Vec+PI model, the weight matrix in the Skip-Gram model is initialized with PubMed word vectors. In our Mut2Vec+R model, the output vectors of the basic Mut2Vec model is post-processed in the retrofitting module. In our Mut2Vec+PI+R model, both the initialization with PubMed word vectors and the post-processing are applied

Our Mut2Vec pipeline is summarized as follows. First, we initialize the weight matrix in embedding lookup layer of Skip-Gram model using word vectors, which is pre-trained on PubMed abstracts. Because we needed initial gene vectors, we selected only gene word vectors from the pre-trained word vectors. Next, we trained the gene-level mutation vectors with the ICGC mutation profiles using the initialized Skip-Gram model. We considered co-occurring gene mutations in a sample as contexts, just like words co-occurring in a sentence are considered as contexts in the NLP domain. Finally, we retrofitted the trained mutation vector on the Protein-Protein Interaction network data of BioGRID. The whole pipeline is described in Fig. 1.

### Skip-Gram model

**Fig. 2:**
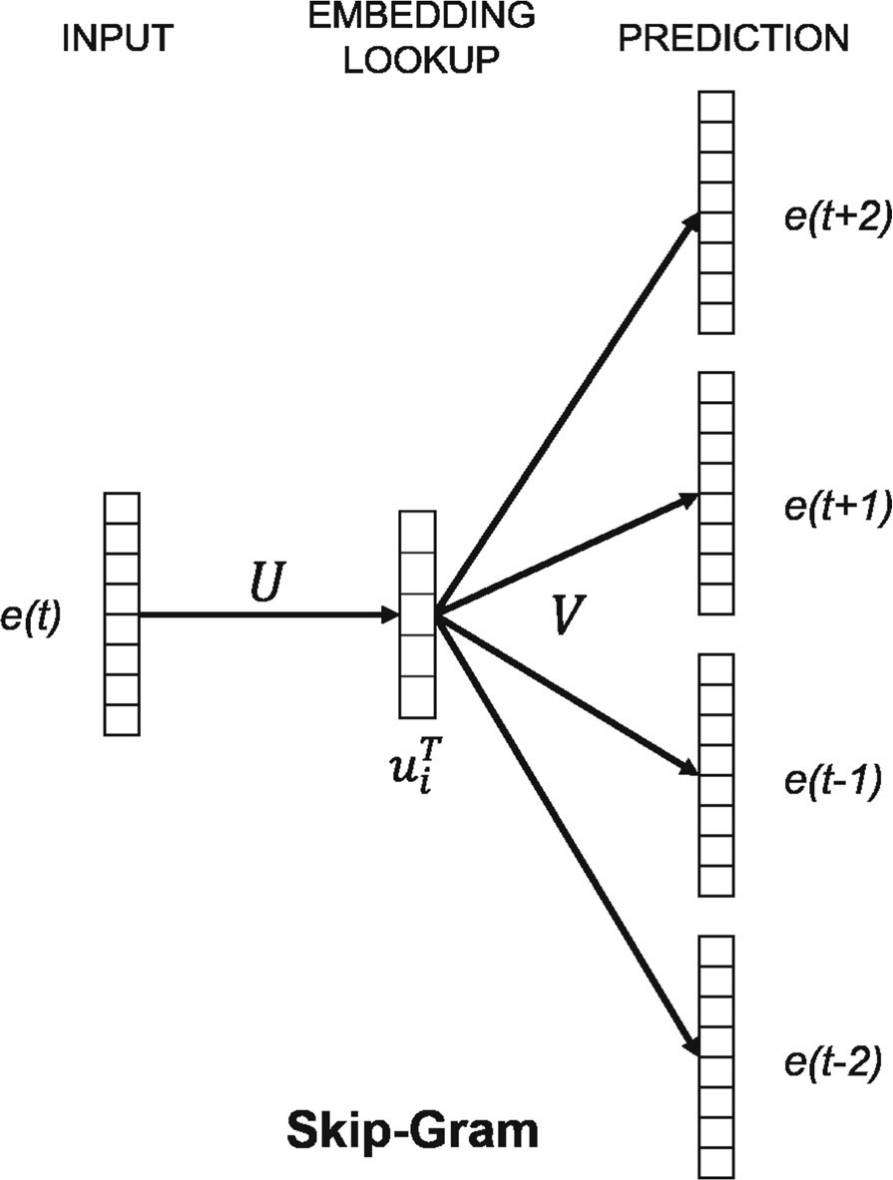
An overview of the Skip-Gram model. The Skip-Gram model consists of an input layer, embedding lookup(hidden) layer, and prediction layer. The result of the embedding lookup layer is the distributed representation of the target word

The Skip-Gram model is a multi-layered neural network, as shown in Fig. 2. The ultimate objective of this model is to correctly predict the surrounding entities based on the entity that is embedded in the network. To achieve this objective, we need to train the model by using our “entity” and its contextual “entities”. The embedded entities are the mutated genes while the output or their contextual entities are the co-occurring mutated genes. Thus, we train the Skip-Gram model in iteration by using mutated genes as input and minimizing the prediction error gap between the output and their co-occurred mutations.

By using the Skip-Gram model, we maximize the probability as follows,

$$\begin{array}{*{20}l}
\begin{aligned} p(C_{i}|e_{i})&=p(c_{1},c_{2},\ldots,c_{k},\ldots,c_{l-1},c_{l}|e_{i})\\ &\approx \prod_{e_{j}\in C_{i}}{p(e_{j}|e_{i})} \end{aligned}\\ &\begin{aligned} (e_{i} \in E \textrm{ and } e_{j}, c_{k}\in C_{i} \subset E) \end{aligned} \end{array} $$

where *C*_*i*_={*c*_1_,*c*_2_,…,*c*_*k*_,…,*c*_*l*−1_,*c*_*l*_} is the context set of an entity *e*_*i*_, context size *l* is |*C*_*i*_|, and *E* is a set of entities to be embedded. When embedding words in text, context size *l* is fixed. However, in our case, it is difficult to fix the context size because the number of mutations in each sample varies. Some samples have less than 10 gene mutations, while others have more than 1000 gene mutations. In addition, since mutations included in a single patient sample are not sorted according to a certain biological order, drawing a mutation vector by shifting the context window is illogical, unlike the case of NLP.

To assign various co-occurring contexts to a mutation, we performed random sampling without replacement on each patient sample 10 times. The size of the random samples was 10. We assumed that patient samples with an excessive number of mutations tend to be highly noisy. Also, we found the information extracted from patient samples with small quantities of mutations was critical for embedding each mutation vector. As we conducted the same random sampling procedure regardless of the mutation quantity of each patient sample, noisy samples with an excessive number of mutations were used less in vector embedding processes. On the other hand, patient samples with small mutation quantities were used frequently.

The conditional probability mentioned above can be expressed with latent parameters of a neural network and a softmax function as below,

$$\begin{array}{*{20}l} p(e_{j}|e_{i})& = \frac{exp\left(u_{i}^{T}v_{j}\right)}{\sum_{k=1}^{|E|}{exp\left(u_{i}^{T}v_{k}\right)}}\\ J(U, V) &= \frac{1}{N}\sum_{i}^{N}\sum_{e_{j}\in C_{i}}{log(p(e_{j}|e_{i}))} \end{array} $$

where *U* is a weight matrix for an embedding lookup layer, $u_{i}^{T}$ is a distributed representation of i-th entity, N is the number of all training entities which can be defined with contexts, (*e*_*i*_,*C*_*i*_). *V* is an output weight matrix, and *v*_*j*_ is j-th row of the matrix *V*. Our goal is to maximize the objective function *J*(*U*,*V*) above.

However, the basic Skip-Gram model described above suffers from high computational cost. Due to the summation calculation in the denominator of *p*(*e*_*j*_|*e*_*i*_), the computational cost for calculating *J*(*U*,*V*) is often high especially for large vocabularies (entities). To address this issue, Mikolov et al. [18] proposed a Skip-Gram model that has an additional feature called negative sampling. Instead of using the softmax function, negative sampling directly uses the sigmoid function *σ*(*x*) to represent each entity’s conditional probability.

1 $$ \begin{aligned} \sigma(x) &= \frac{1}{1+exp(-x)}\\ p(e_{j}|e_{i}) &= \sigma\left(u_{i}^{T}v_{j}\right)\\ p\left(\bar{e_{j}}|e_{i}\right) &= 1-\sigma\left(u_{i}^{T}v_{j}\right) \end{aligned}  $$

Using the re-defined conditional probability above, negative sampling maximizes the objective function *J*_*NEG*_(*U*,*V*) as below

$$\begin{aligned} J_{NEG}(U,V)^{i} &= \sum_{j\in C_{i}}log(p(e_{j}|e_{i}))+\sum_{l\in D_{i}}{log(p(\bar{e_{l}}|e_{i}))}\\ J_{NEG}(U,V) &= \frac{1}{N}\sum_{i=1}^{N}J_{NEG}(U, V)^{i}\\ \left(D_{i}\subset C_{i}^{C}, C_{i}^{C} \right.&\left.= E - C_{i}{\vphantom{C_{i}^{C}}}\right) \end{aligned} $$

where *D*_*i*_ is a sampled subset of $C_{i}^{C}$, which is a complement of *C*_*i*_. Also, |*D*_*i*_| is fixed. The sampling process is done using a distribution of entities raised to the 3/4rd power. In conclusion, the Skip-Gram model equipped with negative sampling maximizes the occurrence probability of contextual entities and minimizes the occurrence probability of non-contextual entities, conditioned on the occurrence of the entity. We used Gensim [19], which is a Python library, for the implementation.

### Retrofitting

As words and phrases have synonyms and paraphrases respectively, Faruqui et al. [14] utilized structured lexical meaning networks, WordNet [20], FrameNet [21] and the Paraphrase database (PPDB) [22], to post-process vectors of entities. The purpose of this post-processing was to ensure the vectors have similar representations if they are synonyms or paraphrases. The processing is done by minimizing the function $J(Q,\widetilde {Q})$ defined by $J_{i}(Q,\widetilde {Q})$ and *J*_(*i*,*j*)_(*Q*) as follows,

$$\begin{array}{*{20}l} J_{i}(Q,\widetilde{Q}) &=& \alpha_{i}||q_{i}-\widetilde{q}_{i} ||^{2} \\
J_{(i,j)}(Q) &=& \beta_{ij}||q_{i}-q_{j} ||^{2} \\
(j \in {S}_{i}, {q}_{i}, {q}_{j}& \in &Q\, \text{and}\, \widetilde{q}_{i} \in \widetilde{Q})
\end{array} $$

where $\widetilde {q_{i}}$ is a trained vector, *q*_*i*_ is a post-processed vector, *S*_*i*_ is a set of entities similar to an entity i, and *q*_*j*_ is a vector of which its entity is similar to an entity i and is included in *S*_*i*_. *α*_*i*_ and *β*_*ij*_ are hyperparameters for each entity and a pair of entities, where *α*_*i*_=1, and *β*_*ij*_=|*S*_*i*_|^−1^. The hyperparameter values were used as the default values for the model. Finally, the objective function can be obtained by the formula below.

$$\begin{array}{*{20}l}  J(Q,\widetilde{Q}) = \sum_{i}^{n} \left (J_{i}(Q,\widetilde{Q}) +\sum_{j \in S_{i}}J_{(i,j)}(Q) \right) \end{array} $$

Likewise, if two gene mutations were involved in the same cellular process, we assumed that they have similar effects, such as malfunctions or abnormal activations, on biological processes. From the BioGRID network, we selected genes one hop apart from a certain gene as similarly functioning genes, and made them similar each other using the retrofitting process described above. Retrofitting codes are available at https://github.com/mfaruqui/retrofitting.

## Results

### Driver/Passenger mutation visualization

Many mutations in a single cancer sample are not entirely related to cancer. The driver mutation directly affects the progression of the cancer, while the passenger mutation does not play any particular role. In fact, driver mutations are common in many cancer cells of patients, while passenger mutations are not [23].

We performed data visualization to see if our mutation vectors reflect the mutual distinction between driver and passenger mutations in the vector space. The driver/passenger mutation information was obtained from the driver mutation database IntOGen [24]. We also conducted k-means clustering on the driver and passenger mutation vectors before reducing dimensions by Principal Component Analysis, and calculated the Normalized Mutual Information(NMI) to assess the clustering result. The NMI is defined as

$$\begin{array}{*{20}l} NMI(\Omega, C) = \frac{MI(\Omega, C)}{[H(\Omega) + H(C)]/2} \end{array} $$

where *Ω*={*ω*_1_,*ω*_2_,...*ω*_*I*_} is the set of cluster labels and *C*={*c*_1_,*c*_2_,…*c*_*J*_} is the set of class labels. In our case, *C*={*driver*,*passenger*}. *MI* is mutual information defined as

$$\begin{array}{*{20}l} MI(\Omega, C) = \sum_{i}\sum_{j}p(\omega_{i},c_{j})log\frac{p(\omega_{i},c_{j})}{p(\omega_{i})p(c_{j})} \end{array} $$

where *p*(*ω*_*i*_), *p*(*c*_*j*_), and *p*(*ω*_*i*_,*c*_*j*_) are the probabilities of a mutation occurring in cluster *ω*_*i*_, class *c*_*j*_, and the intersection of *ω*_*i*_ and *c*_*j*_, respectively.

*H* is entropy defined as

$$\begin{array}{*{20}l} H(\Omega) = -\sum_{i}p(\omega_{i})log(p(\omega_{i})) \end{array} $$

Experiments were performed on three cancer types (CM, BRCA, LUAD) with the highest number of “known” driver mutations among 29 cancer types. According to Table 1, driver mutation data contains far more predicted mutations than known mutations. Passenger mutations are all predicted mutations. Since known driver mutations are more reliable than predicted driver mutations, we used only “known" driver mutations for a more accurate comparison. Also, there are only “predicted” for passenger mutations in the database. Since the number of passenger mutations is much larger than the number of driver mutations, randomly sampled passenger mutations were selected for the visualization process.

**Table 1:** IntOGen data description for three cancer types

	Drivers		Passengers
Type	Known	Predicted	Predicted
BRCA	22	473	13702
CM	29	607	16863
LUAD	23	505	13929

**Fig. 3:**
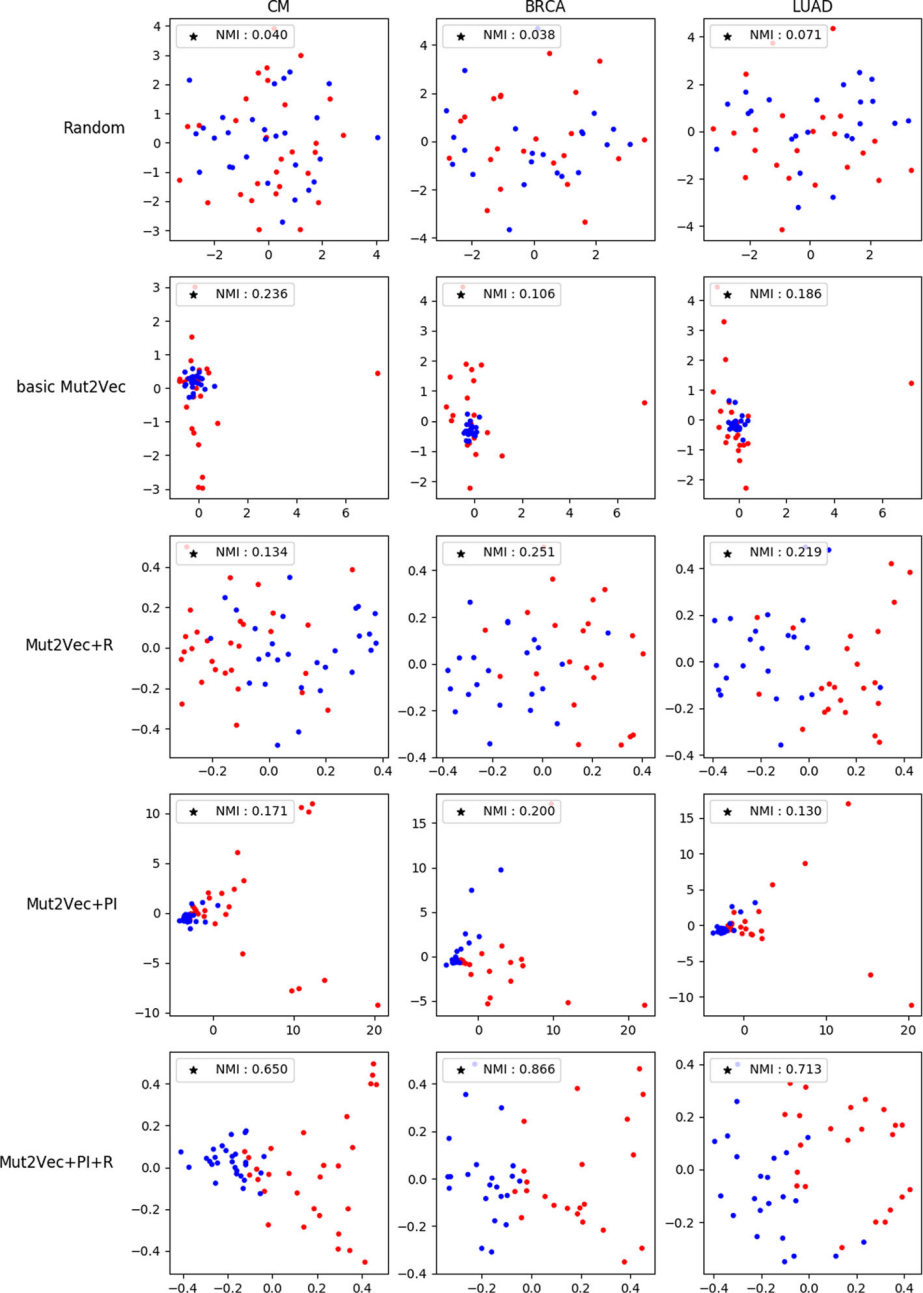
Driver/passenger mutations visualization. Visualization with Principal Component Analysis, shows the clear difference between driver and passenger mutation classes when PubMed information is applied. Red dots represent known driver mutations and blue dots represent sampled predicted passenger mutations. Normalized Mutual Information (NMI) is also calculated based on the results of k-means clustering

We obtained interesting results using our mutation vectors. As shown in Fig. 3, vectors using ICGC dataset perform slightly better than randomly generated vectors, and the mutual distinction between driver mutations and passenger mutations in the vector space became clearer when PPI network knowledge was added. After adding PubMed information, we could confirm that both driver and passenger mutations were properly classified. Furthermore, the improvements measured by NMI support our visualization results. Compared with randomly generated vectors, our mutation vectors improved the NMI scores of all three cancer types. We could observe dramatic performance improvements when both literature information and PPI network information were applied together.

**Fig. 4:**
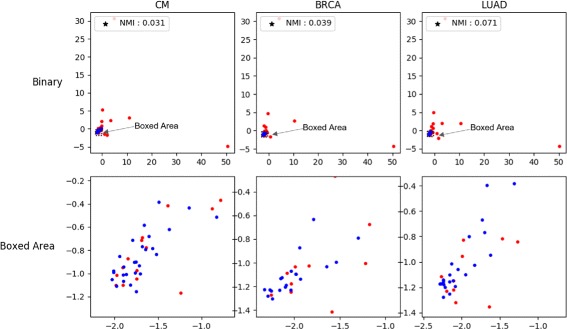
Driver/passenger mutations visualization of binary vectors. Visualization with Principal Component Analysis of binary mutation vectors. The binary vectors are made by selecting column vectors of patient-mutation profiles. Because driver mutations tend to frequently appear in cancer profiles of patients, there are many 1s in the driver mutation vectors and the size of the driver mutation vectors are large. Therefore, some of driver mutation vectors are far from most mutation vectors, The “Boxed Area” is the expanded visualization of the boxed area of “Binary” visualization where most of passenger mutation vectors exist. We found that the drivers and passengers were actually scrambled while they seemed to be well-separated in a broader scope

We also conducted this visualization experiment with binary mutation representation. In the ICGC patient-mutation profile, we defined a binary mutation vector of dimension equal to the number of samples in the ICGC dataset. Each dimension of the vector encodes the existence of a mutation in a corresponding sample. In other words, the binary vector of a mutation has a value of 1 only for the dimension corresponding to the sample that has the mutation; however, the binary vector has a value of 0 for all other dimensions. Figure 4 shows the visualization of binary mutation vectors. The distinction between passenger mutations (blue dots) and driver mutations (red dots) misleadingly seems to be accurate since the blue dots are notably more clustered than the red dots. In fact, driver mutations are more frequently observed in patients than passenger mutations. Therefore, some of the binary vectors of driver mutations are larger and are positioned far from the area where most mutations are clustered. However, when we expanded the area where most passenger mutations existed, we could observe that the drivers and passengers were actually scrambled. The NMI scores of binary representation were also lower than the Mut2Vec+PI+R scores. Binary representation obtained scores of 0.031, 0.039, 0.071 for CM, BRCA, and LUAD, respectively, using NMI, whereas Mut2Vec+PI+R obtained scores of 0.650, 0.866, 0.713 for CM, BRCA, and LUAD, respectively, using NMI. The mutation vectors from Mut2Vec+PI+R model can better represent the information on driver mutations than binary vector representation.

For the comparison, we trained 300-dimensional mutation vectors using an autoencoder [25] and a denoising autoencoder [26], and conducted a visualization experiment. The autoencoder obtained scores of 0.007, 0.074, 0.071 for CM, BRCA, and LUAD, respectively, using NMI. The denoising autoencoder obtained scores of 0.031, 0.040, 0.038 for CM, BRCA, and LUAD, respectively, using NMI. Also, we found that the vectors trained on the autoencoders could not effectively distinguish driver mutations from passenger mutations. The visualization results are listed in Additional file 1.

### Driver mutation identification

Based on our previous visualization, we can infer that driver mutation vectors have their own properties which help distinguish driver mutations from other mutations. From this inference, we clustered the entire set of mutations in vectors from Mut2Vec+PI+R and examined whether the cluster contained many driver mutations.

The k-means algorithm with the option of 200 clusters was applied to the clustering process. Next, we selected the most enriched cluster that contains the most driver mutations, and built a contingency table, which is shown in Table 2. We estimated the statistical significance based on the hypergeometric test [27] on entire driver gene mutations in the IntOGen database. In the database, there were 67 unique “known" drivers and 594 “predicted" drivers, all of which intersect with embedded mutations. In our most enriched cluster, we could find 21 known drivers with a *p*-value of 3.74e-37. Considering both known and predicted driver mutations, we found 45 drivers with a *p*-value of 2.04e-51. The remaining 18 mutations were not referred to as drivers in the database. As the cluster had high statistical significance, we concluded that the mutation vectors from our Mut2Vec+PI+R model captured the characteristics of driver mutations. Other contingency tables of different clustering methods(Agglomerative hierarchical clustering, BIRCH, Spectral clustering, Affinity propagation, and Gaussian mixture) that performed based on different numbers of clusters(50, 100, 300, and 500) are listed in Additional file 2.

**Table 2:** The most enriched cluster characterized using mutation vectors from Mut2Vec+PI+R

Labels	In cluster	In population	*p*-value
Known	21	67	3.74e-37
Known+predicted	45	661	2.04e-51
Candidates	18	17923	
All	63	18584	

We also performed an enrichment analysis on the KEGG PATHWAY database [28] using Fisher’s exact test. We used a publicly available enrichment analysis platform, which was given by Enrichr [29]. The *p*-value was adjusted using the Benjamini-Hochberg method for correcting multiple hypotheses testing. As Table 3 shows, “Pathways in cancer" was the most enriched pathway with an adjusted *p*-value of 9.98e-24 and 25 overlapped genes. All the five pathways were related to cancer or the general characteristics of cancer such as metabolism and misregulation, since various types of driver mutations were grouped in the most enriched cluster.

**Table 3:** Pathway enrichment analysis of the most enriched cluster characterized using mutation vectors from Mut2Vec+PI+R

KEGG PATHWAY	Adjusted *p*-value	Overlap
Pathways in cancer	9.98e-24	25/397
Central carbon metabolism in cancer	2.51e-22	15/67
Transcriptional misregulation in cancer	6.76e-19	17/180
MicroRNAs in cancer	6.27e-14	16/297
Prostate cancer	1.93e-13	11/89

Based on the above observation, we carried out a further experiment. We hypothesized that if most of the mutations gathered in a cluster are drivers, the unidentified mutations in the cluster are most likely to be driver mutations. To test our assumption, we investigated mutations in the most enriched cluster, as shown in Table 4. We focused mainly on retrieving information on the 18 candidate mutations which were not identified as drivers in the IntOGen database.

**Table 4:** Genes in the most enriched cluster characterized using mutation vectors from Mut2Vec+PI+R

Known	Predicted	Candidate
ABL1	ASXL1	BCL2
ALK	BAP1	CISH
DNMT3A	BCL6	CRLF2
EGFR	CALR	DUSP22
ERBB2	CCND1	ERG
FGFR2	CEBPA	EWSR1
FGFR3	ETV6	FHIT
FLT3	FGFR1	MAML2
GNAQ	H3F3A	MYBL1
HRAS	IKZF1	MYCL
IDH2	MET	PDGFRB
JAK2	MYCN	PLAG1
KIT	NF2	PRKACA
MYC	NOTCH1	SPINK1
MYD88	NTRK1	SS18
NPM1	PAX5	TERT
NRAS	PDGFRA	TFE3
RB1	PPM1D	TP63
RUNX1	RET	
SDHB	RHOA	
SMO	ROS1	
	SMARCB1	
	TET2	
	WT1	

Through searching entire recently published biomedical papers since 2015, we found out that 11 of the candidate mutations were reported as driver mutations. Table 5 shows the literature search results. BCL2 is an important driver of leukemia and referred to as a driver mutation [30]. ERG is reported to be a driver of carcinogenesis in prostate cancer [31, 32]. The loss of fragile histidine triad protein (FHIT) is strongly related to pancreatic ductal adenocarcinomas [33]. MAML2-MECT1 fusion is a driver in salivary gland and bronchial gland mucoepidermoid carcinoma [34, 35]. MYBL1 is a driver of adenoid cystic carcinoma when related to MYB [36, 37]. As a member of the myelocytomatosis oncogene family, MYCL is a driver oncogene of lung carcinoma [38, 39]. PDGFRB was recently reported as a driver of the majority of sporadic infantile and adult solitary myofibromas [40]. PRKACA was identified by recent sequencing as a driver of cortisol-producing adenomas and perihilar cholangiocarcinoma [41, 42]. SS18 with SSX fusion has been reported as driver of synovial sarcoma in many research studies [43–47]. TERT has also been reported as a cancer driver of various tissues including thyroid and liver [48–57]. Variation in TP63 is associated with drivers of squamous cell lung cancer [58, 59].

**Table 5:** Literature search results on driver candidates

Gene	Tissue	References	
BCL2	Leukemia	[30]	
ERG	Prostate	[31, 32]	
FHIT	Pancreas	[33]	
MAML2	Bronchial Glands		
	Salivary Glands	[35]	
MYBL1	Gland	[36]	
MYCL	Lung	[38, 39]	
	Nerve Tissue	[39]	
PDGFRB	Myofibroma	[40]	
PRKACA	Cortisol	[41]	
	Liver	[42]	
SS18	Diathrosis	[43–45]	
TERT	Liver	[48, 52, 54]	
	Melanocyte	[49]	
	Thyroid	[50, 53, 55]	
	Unknown	[51, 57]	
TP63	Squamous Cell	[58, 59]	

We also analyzed candidates that cannot be found in the current literature. Among them, CRLF2 is one of the receptors in the JAK-STAT signaling pathway. This gene is located on the upper part of the pathway, so it affects the overall JAK-STAT pathway by JAK regulation. Activated JAK enhances the dimerization of STAT proteins, and STAT dimers regulate the transcription of downstream proteins that affect cell fate decisions [60]. The overexpression of CRLF2 has already been reported to be associated with acute lymphoblastic leukemia [61]. Similarly, activating mutations of CRLF2 may trigger overactivation of the JAK-STAT signaling pathway, and the activations may influence cell fate decisions. EWSR1 is an RNA binding protein and TFE3 is a transcription regulator. Both genes have already been reported to cause transcriptional misregulation in cancer when fused with other proteins [62–66]. Also, the overexpression of PLAG1 was reported as a driver event of T-cell acute lymphoblastic leukemia [67]. Finally, the DUSP22 gene fusion was reported to be related to anaplastic large-cell lymphoma [68].

It shows our Mut2Vec+PI+R can discover potential drivers that are not listed in the public driver mutation database by capturing the characteristics of driver mutations. By clustering in the whole mutation space, we found a cluster of statistically significant driver mutations and consider other mutations as driver candidates while excluding known driver mutations. In our literature search, we found articles which some candidates are referred to as actual driver mutations. To our surprise, some of them were recently discovered as driver mutations, which shows our vector captured recently confirmed driver mutations in literatures only with the application of the Skip-Gram model and retrofitting process.

We could make this important discovery that our vector captures novel driver mutations because our vector learned the mutation context from the recent PubMed articles and learned the differential characteristics of driver mutations in patient profiles. Therefore, we repeated the clustering method and literature search on candidate driver mutations of the most enriched cluster resulted from the clustering results using the new vectors from Mut2Vec+PI+R model that was applied only to articles published from past until 2015, to see if our Mut2Vec pipeline could find novel driver mutations just by this simple clustering method. We call this model which limits the PubMed information Mut2Vec+PI(until2015)+R.

As Table 6 shows, the most enriched cluster with known drivers obtained from the clustering results using Mut2Vec +PI(until2015)+R. The table shows that Mut2Vec+PI (until2015)+R still had statistically significant *p*-values(1.21e-8 and 3.99e-28) in the case of known and entire driver mutations. Among the candidate mutations, we discovered two novel driver mutations which were published after 2015, as listed in Table 7. The missense mutations in RAD51 could be drivers of lung and kidney cancers and metastatic diseases [69]. RAD51AP1 was identified as a potential driver of melanoma metastasis [70]. This finding implicates that the mutation vector generated by our Mut2Vec pipeline can be used to find novel driver mutations.

**Table 6:** Statistics of the most enriched cluster characterized using mutation vectors from Mut2Vec+PI(until2015)+R

Labels	In cluster	In population	*p*-value
Known	8	67	1.21e-8
Known+predicted	40	661	3.99e-28
Candidates	81	17923	
All	121	18584

**Table 7:** Literature search results on driver candidates characterized using mutation vectors from Mut2Vec+PI(until2015)+R

Gene	Date	Tissue	References
RAD51	2016	Lung, Kidney	[69]
RAD51AP1	2017	Melanoma	[70]

## Discussion

### Improvement with existing information

In this study, we attempted to integrate existing biomedical literature and protein-protein interaction network into mutation representations. The above two experiments show that by applying knowledge from different data sources, we can achieve quality improvements of distributed representations of mutations.

We achieved a clearer distinction between driver and passenger mutations in visualization when the vectors were pre-trained on PubMed information. In the biomedical literature, the surrounding context of driver and passenger mutations is distinguishable. For example, words such as “critical”, “drug”, “resistant” or “cancer progression” co-occur more frequently with gene names of driver mutations than with gene names of passenger mutations. In addition, driver mutations tend to appear simultaneously in a sentence or a paragraph. This contextual difference between driver and passenger mutations is considered when pre-training mutation vectors on PubMed information.

After the BioGRID information was applied, the visualization results and NMI scores improved. The mutations of the interacting genes (proteins) are adjusted by retrofitting so they are close to each other. Although some mutation vectors are incorrectly trained and become outliers by the noise information in PubMed and ICGC, they can be corrected using the biological knowledge. In this work, we utilized BioGRID. However, due to the availability of many other PPI networks, comparison with those networks using our pipeline seems viable. Also, it is important to consider negative interaction information [71] regarding the absence of interaction between two proteins. However, as the retrofitting procedure takes only positive relations into account, we were unable to incorporate the negative interaction information in our current pipeline, which we leave for future work.

After applying the biomedical literature and interaction information, we clustered the resulting vectors. We extracted the driver mutation candidates from a cluster which was statistically enriched with the known driver genes according to IntOGen, and found studies that reported some of the candidates to be drivers. In this work, we conducted an enrichment test using all known drivers of the database regardless of the cancer types. To provide the cancer-type-specific driver candidates, we also conducted several enrichment tests using the known drivers of each cancer type in the database. However, the most enriched clusters with known drivers of each cancer type were actually identical to those with all known drivers regardless of cancer type. To resolve this issue, we produced cancer-type-specific embeddings with only the ICGC samples corresponding to each cancer type. However, the size of divided sample became too small to train reliable mutation vectors, compared with the size of entire ICGC sample. Moreover, the PubMed initialization process provided general biological contexts, and the retrofitting process fixed the incorrectly embedded vectors using PPI information which is constructed with general human biological process, not with the biological process of specific tissue. Thus, the representations tend to be general rather than cancer-specific especially when the amount of cancer-type-specific data is insufficient. We leave the task of training the cancer-type-specific mutation representations for future work.

### Application of Mut2Vec

Mut2Vec, our proposed pipeline for distributed representations of mutations, can be used for various purposes. Unlike the conventional binary representation, Mut2Vec contains mutation-specific properties in the continuous vector. We demonstrated that it can be used to identify potential driver mutations.

It can also be utilized as the properties of each mutations for an analysis of disease or of patient characteristics. After evaluating the results of the above clustering experiment from Mut2Vec+PI+R, we found that other clusters besides the driver cluster also had their own characteristics. Table 8 shows the top 10 clusters from the enrichment analysis of the KEGG PATHWAY. The result shows that each of our mutation vectors has its own functional characteristics, and the mutation vectors with a similar function are grouped in the vector space with strong statistical significance. The enrichment analysis of KEGG PATHWAY shows that Mut2Vec considers genetic functional similarity as well as mutational similarity.

**Table 8:** Top 10 enriched clusters in KEGG PATHWAY

KEGG PATHWAY	Adjusted *p*-value	Overlap	Cluster size
Neuroactive ligand-receptor interaction	2.22e-63	47/277	85
Chemical carcinogenesis	9.37e-52	26/82	38
Cytokine-cytokine receptor interaction	2.48e-46	36/265	72
Metabolic pathways	1.80e-45	58/1239	89
Ribosome	4.50e-40	27/265	35
Cell cycle	5.55e-37	24/137	37
Endocytosis	6.75e-34	25/277	32
Complement and coagulation cascades	8.25e-33	21/124	35
Fanconi anemia pathway	6.41e-28	36/259	146
Systemic lupus erythematosus	4.26e-27	19/265	20

Additionally, Mut2Vec can be used to construct cancer or patient vectors. However, it is difficult to represent the continuous cancer vector as a summation of our continuous mutation vectors. Since the significance of each driver mutation varies depending on the cancer type and our embedding pipeline does not consider the summation of mutation vectors when representing a cancer vector, the mutation information can be unclear due to the sum operation. In the NLP domain, a sentence vector is not generated by a sum of word vectors. Word vectors are used as features in a deep learning model and a sentence vector is produced implicitly in the model. Likewise, a cancer vector can be generated implicitly when using our Mut2Vec in deep learning tasks such as cancer subtype classification or drug sensitivity prediction.

## Conclusions

In this work, we proposed Mut2Vec, a novel pipeline, for training a distributed representation of mutations on ICGC genetic mutation profiles of cancer donors. To compensate for the incompleteness and noise in the raw data, we augmented our model using PubMed data and the BioGRID protein-protein interaction network. In the visualization of driver and passenger mutation vectors, we showed that our vector determined whether a mutation was a driver or passenger. We also identified driver mutation candidates by investigating the most enriched cluster with known driver mutations after clustering the entire mutation vectors. We confirmed the validity of driver candidates with recent literature in which true driver mutations are reported.

We expect Mut2Vec to benefit researchers in many applications such as patient classification and drug response prediction. We also hope our discovery will assist many research projects with insufficient dataset in training embedding vectors.

The pre-trained mutation vectors and the candidate driver mutations are available at http://infos.korea.ac.kr/mut2vec.

## Additional files

Additional file 1 It contains the visualization results with mutation vectors trained with an autoencoder and a denoising autoencoder. (PDF 427 kb)

Additional file 2 It contains the most enriched clusters with IntOGen driver mutations obtained by six clustering methods(K-Means, Agglomerative hierarchical clustering, BIRCH, Spectral clustering, Affinity Propagation, and Gaussian Mixture) and five options of the number of clusters(50, 100, 200, 300 and 500); except Affinity Propagation. (PDF 108 kb)

## Abbreviations

BRCA: Breast cancer
CM: Cutaneous melanoma
ICGC: International Cancer Genome Consortium
KEGG: Kyoto encyclopedia of genes and genomes
LUAD: Lung adenocarcinoma
Mut2Vec: Mutation vectors trained with Skip-Gram
Mut2Vec+R: Mutation vectors trained with Skip-Gram then processed with BioGRID data
Mut2Vec+PI: Mutation vectors initialized with PubMed literature vectors then trained with Skip-Gram
Mut2Vec+PI+R: Mutation vectors initialized with PubMed literature vectors, trained with Skip-Gram then processed with BioGRID data
NLP: Natural language processing
PPI: Protein-protein interaction
