# Supplementary material for: Mut2Vec: distributed representation of cancerous mutations
Source: BMC Med Genomics. 2018 Apr 20;11(Suppl 2):33. doi: 10.1186/s12920-018-0349-7 (PMC5918431; doi:10.1186/s12920-018-0349-7)
Supplement: Supplementary file 1 — It contains the visualization results with mutation vectors trained with an autoencoder and a denoising autoencoder. (PDF 427 kb) [file 12920_2018_349_MOESM1_ESM.pdf]

Mut2Vec: distributed representation of cancerous mutations Additional File 1

Sunkyu Kim, Heewon Lee, Keonwoo Kim, and Jaewoo Kang

This file lists the visualization results with mutation vectors trained with an autoencoder[1] and a denoising autoencoder[2]. In both autoencoders, we used an Adagrad optimizer[3] for a learning rate of 0.1 and trained it by 500 epochs.

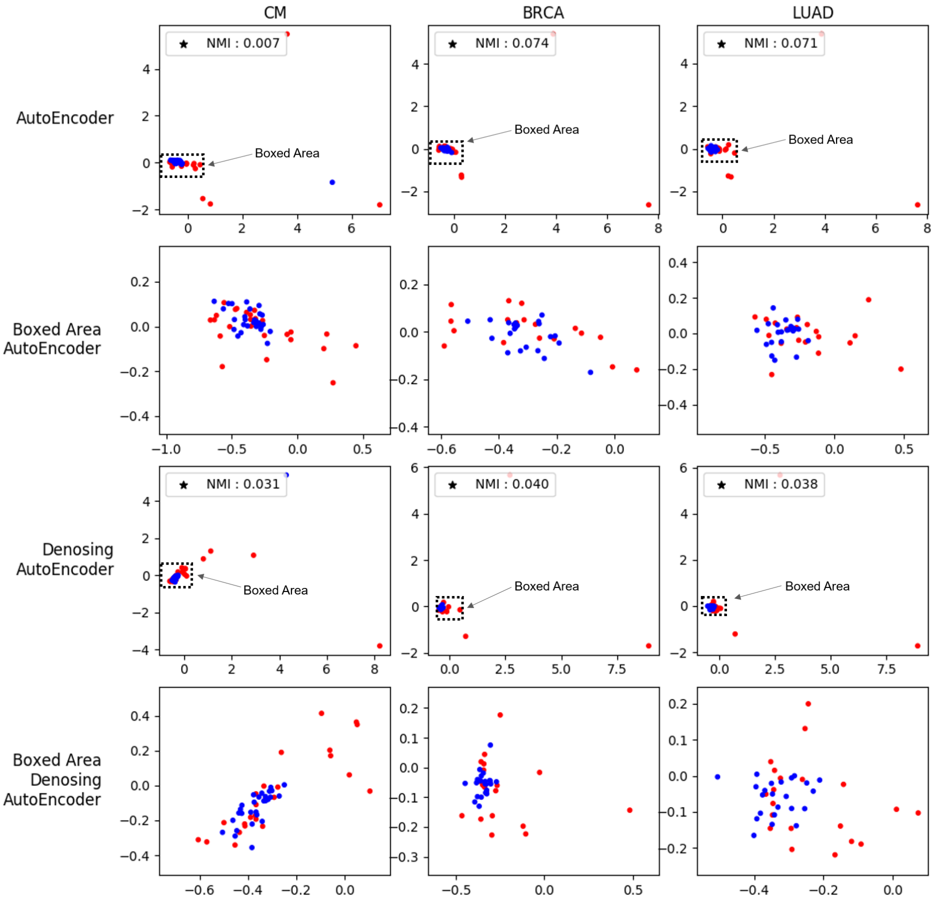

References

[1] LeCun, Y., Bottou, L., Bengio, Y., Haffner, P.: Gradient-based learning applied to document recognition. Proceedings of the IEEE **86**(11), 2278–2324 (1998)

[2] Vincent, P., Larochelle, H., Bengio, Y., Manzagol, P.-A.: Extracting and composing robust features with denoising autoencoders. In: Proceedings of the 25th International Conference on Machine Learning, pp. 1096–1103 (2008). ACM

[3] Duchi, J., Hazan, E., Singer, Y.: Adaptive subgradient methods for online learning and stochastic optimization. Journal of Machine Learning Research **12**(Jul), 2121–2159 (2011)
