# Supplementary material for: Mut2Vec: distributed representation of cancerous mutations
Source: BMC Med Genomics. 2018 Apr 20;11(Suppl 2):33. doi: 10.1186/s12920-018-0349-7 (PMC5918431; doi:10.1186/s12920-018-0349-7)
Supplement: Supplementary file 2 — It contains the most enriched clusters with IntOGen driver mutations obtained by six clustering methods(K-Means, Agglomerative hierarchical clustering, BIRCH, Spectral clustering, Affinity Propagation, and Gaussian Mixture) and five options of the number of clusters(50, 100, 200, 300 and 500); except Affinity Propagation. (PDF 108 kb) [file 12920_2018_349_MOESM2_ESM.pdf]

Mut2Vec: distributed representation of cancerous mutations Additional File 2

Sunkyu Kim, Heewon Lee, Keonwoo Kim, and Jaewoo Kang

This file lists the most enriched clusters resulting from the clustering of Mut2Vec+PI+R mutation vectors, which was conducted using six clustering methods(affinity propagation, agglomerative hierarchical clustering, BIRCH, Gaussian mixture, spectral clustering, and k-means clustering) with five different cluster numbers(50, 100, 200, 300, and 500, except for affinity propagation). To provide more candidates, we dropped clusters of which all contents were annotated as drivers(known or predicted) in the IntOGen[1] database. We utilized scikit-learn[2] with default settings.

1. Affinity Propagation

We do not set the number of clusters when conducting affinity propagation. When using the default settings of scikit-learn, the resulting number of clusters was 1036.

The number of clusters was 1036, the p-value was 3.625e-13.

Label	Mutation
Known	CSF3R GNA11 GNAQ HRAS MAP2K1 NRAS
Predicted	ARAF H3F3A
Candidate	GNA14 PDGFRB

2. Agglomerative Hierarchical Clustering

The number of clusters was 50, the p-value was 5.076e-20.

Label	Mutation
Known	ABL1 ALK BAX EGFR ERBB2 FGFR2 FGFR3 FLT3 JAK2 KIT MYC MYD88 RB1 RUNX1 SMO
Predicted	BAP1 BCL11A BCL6 BIRC3 BTK CCND1 CD79A CD79B ERCC1 ETV6 FGFR1 FGFR4 GATA1 GATA2 IKZF1 JAK1 JAK3 MET MGMT MYCN NOTCH1 NUP98 PAX5 PDGFRA PRDM1 RET ROS1 SMARCB1 SYK USP6 WWOX
Candidate	ASS1 BAALC BAD BAG3 BCL2 BCL3 BLNK CASP3 CBX2 CCNE1 CRLF2 DUSP22 EML4 ETS2 ETV1 ETV4 EWSR1 FHIT FLI1 FPGS GNMT HMGA2 HOXA1 IGF1R ITK KLF1 KLLN LCK LMO2 LYL1 MAML2 MCL1 MDM2 MDM4 MEIS1 MGEA5 MN1 MTAP MYBL1 NCOA2 NKX2-1 ORAOV1 PBX1 PBX3 PDCD5 PDSS2 PHF1 PLAG1 RARA RRM1 SHMT2 SPIB SS18 TAL1 TCL1A TERT TFE3 TK1 TLX1 TLX3 TOP2A TP63 TYMS XIAP ZAP70 ZNF217

The number of clusters was 100, the p-value was 3.310e-20.

Label	Mutation
Known	DICER1 DNMT3A GNA11 GNAQ GNAS HRAS IDH2 MAP2K1 MAP2K2 NF1 NPM1 SDHB STK11 U2AF1 VHL
Predicted	ARAF ASXL1 BCOR CALR CEBPA CREBBP CSNK1A1 DIS3 EIF1AX FBXW7 FLCN H3F3A KAT6B MED12 MEN1 NF2 NSD1 PDGFRL PHF6 PIK3R2 PIK3R3 POLE PPP2R1A PRKAR1A PTCH1 RASA1 RHOA SDHA SDHAF2 SDHC SDHD SMARCE1 SRSF2 SUFU TET2 TMEM127 TSC1 TSC2 ZRSR2
Candidate	ARX AUTS2 BTD CC2D1A CDKL5 CHD7 COL4A5 COL7A1 CSF2RA EHMT1 ESCO2 FBN1 FOXG1 GLI2 GLI3 GNA14 GRN HNRNPA0 IMMP2L KRT36 LMNB1 MBD5 MECP2 MID1 MKRN3 MUT NFIX NPHP1 NTRK3 PKD1 PKD2 PLP1 PMP22 POLD1 PORCN PQBP1 PRKACA PRKRA RAB3GAP1 RAI1 RBM20 RMND1 SGCE SHANK3 SIM1 SPAST STX16 STXBP1 SYNGAP1 TBX22 TCOF1 TNFRSF14 YWHAE ZBTB24 ZNF277

The numbers of clusters were 200 and 300, the p-value was 6.859e-20.

Label	Mutation
Known	DNMT3A GNA11 GNAQ HRAS IDH2 MAP2K1 MAP2K2 NPM1 SDHB STK11 U2AF1
Predicted	ARAF ASXL1 CALR CEBPA EIF1AX H3F3A PIK3R2 PIK3R3 POLE SDHA SDHAF2 SDHC SDHD SRSF2 TET2 TMEM127 ZRSR2
Candidate	GNA14 MUT POLD1 PRKACA

The number of clusters was 500, the p-value was 9.061e-18.

Label	Mutation
Known	BRCA1 BRCA2 MLH1 MSH2 MSH6 MUTYH PMS2 RAD51C
Predicted	CHEK2 PALB2 RAD51D
Candidate	RAD51B

3. BIRCH

The number of clusters was 50, the p-value was 6.473e-69.

Label	Mutation
Known	ABL1 ALK BRCA1 BRCA2 CDK4 CDKN2A CSF3R DICER1 EGFR ERBB2 FGFR2 FGFR3 FLT3 GNA11 GNAI2 GNAQ HRAS IDH2 JAK2 KIT KRAS MAP2K1 MLH1 MSH2 MSH6 MUTYH MYC MYD88 NPM1 PMS2 PTPN11 RAD51C RB1 SDHB SF3B1 SMAD4 SMO STK11 TP53 U2AF1
Predicted	ARAF ASXL1 BAP1 BCL6 BIRC3 CALR CCND1 CDC73 CDKN1B CDKN2B CDKN2C CEBPA CHEK2 CSNK1A1 DIS3 EIF1AX ETV6 FBXW7 FGFR1 FGFR4 H3F3A IKZF1 MET MYCN MYOD1 NF2 NUP98 PALB2 PAX5 PDGFRA PIK3CB PIK3R2 PPP2R1A PTCH1 RAD51D RASA1 RET RHOA ROS1 SDHAF2 SDHC SDHD SETBP1 SMARCB1 SRSF2 TMEM127 USP6 WWOX ZRSR2
Candidate	ARMC5 BAALC BCL2 CCNE1 CDKN1C CISH CRKL CRLF2 DLX3 DUSP22 ELANE EML4 EPCAM ETV1 ETV4 EWSR1 EYA4 FHIT GATA5 GLI2 GLI3 GNA14 GRB7 HOXA1 HOXB5 IGF1R KLLN LMO2 MAML2 MDM2 MDM4 MGEA5 MN1 MXI1 MYBL1 NCOA2 ORAOV1 PHF1 PLAG1 PORCN PRKACA PRKCI PSMD10 RACGAP1 RARA SPRY1 SS18 TACSTD2 TCF3 TERT TFE3 TOP2A TP73 TUBB3 WISP3 ZBTB7A ZNF217

The numbers of clusters were 100 and 200, the p-value was 1.720e-54.

Label	Mutation
Known	BRCA1 BRCA2 CDKN2A CSF3R DICER1 FLT3 GNA11 GNAQ HRAS IDH2 JAK2 KRAS MAP2K1 MLH1 MSH2 MSH6 MUTYH MYD88 NPM1 PMS2 PTPN11 RAD51C RB1 SDHB SF3B1 SMAD4 SMO STK11 TP53 U2AF1
Predicted	ARAF ASXL1 BAP1 CALR CDC73 CDKN1B CDKN2B CDKN2C CEBPA CHEK2 CSNK1A1 DIS3 EIF1AX FBXW7 H3F3A MYOD1 NF2 PALB2 PIK3CB PIK3R2 PPP2R1A PTCH1 RAD51D RASA1 RHOA SDHAF2 SDHC SDHD SETBP1 SMARCB1 SRSF2 TMEM127 WWOX ZRSR2
Candidate	ARMC5 CDKN1C DLX3 ELANE EPCAM EYA4 FHIT GATA5 GLI2 GLI3 GNA14 KLLN PORCN PRKACA PRKCI TACSTD2 TERT TP73 TUBB3 WISP3 ZBTB7A

The numbers of clusters were 300 and 500, the p-value was 9.854e-28.

Label	Mutation
Known	CSF3R FLT3 GNA11 GNAQ HRAS IDH2 JAK2 MAP2K1 MYD88 NPM1 PTPN11 SDHB SF3B1 STK11 U2AF1
Predicted	ARAF ASXL1 CALR CEBPA CSNK1A1 DIS3 EIF1AX FBXW7 H3F3A PIK3CB PIK3R2 PPP2R1A RHOA SDHAF2 SDHC SDHD SETBP1 SRSF2 TMEM127 ZRSR2
Candidate	ELANE GNA14 PRKACA PRKCI

4. Gaussian Mixture

The number of clusters was 50, the p-value was 5.947e-52.

Label	Mutation
Known	ABL1 AKT1 ALK CDKN2A CSF3R DICER1 DNMT3A EGFR ERBB2 FGFR2 FGFR3 FLT3 GNA11 GNAQ GNAS HRAS IDH2 JAK2 KIT MAP2K1 MYD88 NF1 NPM1 NRAS PMS2 PTEN PTPN11 RB1 RUNX1 SDHB SMO STK11 U2AF1 VHL
Predicted	ACVR1 ARAF ASXL1 AXIN2 BAP1 BCOR BMPR2 BTK CALR CD79B CDC73 CDKN1B CEBPA CHEK2 CREBBP CSF1R ETV6 FBXW7 FGFR1 FMR1 FOXL2 GATA1 GATA2 H3F3A IKZF1 IRF6 MED12 MEN1 NF2 NOTCH1 NSD1 NTRK1 PAX5 PDGFRA PHOX2B PPM1D PRKAR1A PTCH1 RAF1 RASA1 RET RHOA ROS1 SETBP1 SMARCB1 SMARCE1 SRSF2 SUFU TCF4 TET2 TSC1 TSC2 WT1
Candidate	AIRE AKT3 ALX4 AMELY ARX BCL11B BMPR1A C9orf72 CDKL5 CDKN1C CRLF2 DLX3 DMD DUSP22 EWSR1 EYA1 EYA4 FHIT FOXC1 FOXE1 FOXF1 FOXG1 FOXP2 FZD4 GATA6 GFI1B GLI2 GRN HESX1 HEY2 HNF1B HNRNPA0 HOXB13 HOXD13 IHH JAG1 KLF1 KLLN LHX3 LMNB1 LRP5 LZTR1 MECP2 MSX1 MYBL1 NFIX NKX2-1 NKX2-5 NOG NOTCH3 NR5A1 OTX2 PARK2 PAX2 PAX3 PAX6 PAX8 PAX9 PDCD10 PDGFRB PITX2 PKD1 PKD2 PLAG1 PRKACA PROP1 PTF1A RARA RIT1 SHANK3 SHOX SIM1 SOX10 SOX3 SPINK1 SPRED1 TBX1 TBX5 TERT TFE3 THRB TNFRSF14 TP63 TUBB3 WISP3 WNT10A WNT10B ZIC2 ZNF277

The number of clusters was 100, the p-value was 2.591e-42.

Label	Mutation
Known	ABL1 AKT1 ALK BAX CDK4 DNMT3A EGFR ERBB2 FGFR2 FGFR3 FLT3 GNAQ HRAS IDH2 JAK2 KIT MAP2K1 MYC MYD88 NPM1 NRAS PTEN RB1 SDHB SMO
Predicted	ARAF ASXL1 BAP1 BCL6 BIRC3 BTK CALR CCND1 CD79B CDKN1B CEBPA CSF1R EPHA2 ETV6 FBXW7 FGFR1 H3F3A IKZF1 MET MYCN NF2 NOTCH1 NTRK1 PAX5 PDGFRA PPM1D PTCH1 RAF1 RET RHOA ROS1 SMARCB1 STAT5B TET2 TSC2 WT1
Candidate	AKT3 AURKA BCL2 CCNE1 CISH CRKL CRLF2 DUSP22 ETV1 EWSR1 IGF1R MAML2 MDM2 MIB1 MYBL1 MYCL PAX2 PAX8 PDGFRB PLAG1 PRKACA REL SPINK1 SS18 TERT TFE3 TOP2A

The number of clusters was 200, the p-value was 1.728e-42.

Label	Mutation
Known	ABL1 AKT1 ALK CSF3R DNMT3A EGFR ERBB2 FGFR2 FGFR3 FLT3 GNA11 GNAQ HRAS IDH2 JAK2 KIT MAP2K1 MYD88 NPM1 NRAS PTPN11 SMO
Predicted	ARAF ASXL1 BTK CALR CEBPA CSF1R EIF1AX FBXW7 FGFR1 H3F3A IKZF1 MET MYCN NOTCH1 NTRK1 PDGFRA RET RHOA ROS1 SETBP1 TET2
Candidate	CRLF2 PDGFRB PRKACA RIT1 SPINK1 TERT

The number of clusters was 300, the p-value was 4.445e-24.

Label	Mutation
Known	ALK DNMT3A EGFR ERBB2 FGFR2 FGFR3 FLT3 JAK2 KIT MYD88 NPM1 SMO
Predicted	BTK CALR CEBPA FGFR1 MET MYCN NOTCH1 PDGFRA RET TET2 WT1
Candidate	IGF1R

The number of clusters was 500, the p-value was 1.154e-20.

Label	Mutation
Known	ALK CDK4 EGFR ERBB2 FGFR2 FGFR3 FLT3 JAK2 KIT MYC SMO
Predicted	CCND1 FGFR1 FGFR4 MET MYCN NOTCH1 PPM1D ROS1
Candidate	AURKA AXL CCNE1 CRKL IGF1R MDM2 MDM4 TOP2A ZNF217

5. K-Means Clustering

The number of clusters was 50, the p-value was 2.241e-65.

Label	Mutation
Known	ALK BRCA1 BRCA2 CDKN2A CSF3R DICER1 DNMT3A EGFR ERBB2 FGFR2 FGFR3 FLT3 GNA11 GNAQ GNAS HRAS IDH2 JAK2 KIT MAP2K1 MLH1 MSH2 MSH6 MUTYH MYC MYD88 NF1 NPM1 NRAS PMS2 PTPN11 RAD51C RB1 SDHB SMO STK11 U2AF1 VHL
Predicted	ACVR1 ARAF ASXL1 AXIN2 BAP1 BCOR BIRC3 BMPR2 CALR CCND1 CD79B CDC73 CDKN1B CEBPA CHEK2 CREBBP EIF1AX ETV6 FBXW7 FGFR1 FOXL2 H3F3A IKZF1 IRF6 MED12 MEN1 MYCN NF2 NOTCH1 NSD1 NTRK1 PALB2 PDGFRA PHF6 PHOX2B POLE PRKAR1A PTCH1 RASA1 RET RHOA ROS1 SETBP1 SMARCB1 SMARCE1 SPOP SRSF2 SUFU TET2 TMEM127 TSC1 TSC2 WT1
Candidate	AURKC BCL2 BMPR1A CCNE1 CDKL5 CDKN1C CHD7 CRLF2 DUSP22 ERG EWSR1 FHIT FLNA FOXG1 GFI1B HNF1B HNRNPA0 HOXB13 IHH KLLN LRP5 LZTR1 MECP2 MYBL1 MYCL NOTCH3 PAX2 PAX8 PDCD10 PDGFRB PKD1 PKD2 PLAG1 POLD1 PRKACA RIT1 SHOX SPINK1 SPRED1 TERT TFE3 TNFRSF14 TP63 TUBB3 WISP3 YWHAE

The number of clusters was 100, the p-value was 1.210e-43.

Label	Mutation
Known	ABL1 ALK BRCA1 BRCA2 CDK4 CDKN2A DNMT3A EGFR ERBB2 FGFR2 FGFR3 FLT3 IDH2 JAK2 KIT MLH1 MSH2 MSH6 MYC MYD88 NPM1 PMS2 RB1 SDHB
Predicted	BAP1 CALR CCND1 CEBPA ETV6 FGFR1 IKZF1 MET MGMT MYCN NF2 NOTCH1 PDGFRA RET RHOA ROS1 SMARCB1 TET2 TSC1 TSC2 WT1 WWOX
Candidate	AURKA BCL2 CCNE1 CISH CRLF2 DUSP22 ERG EWSR1 FHIT MDM2 MDM4 MTAP MYBL1 PDGFRB PLAG1 SPINK1 TERT TFE3 TOP2A TUBB3

The number of clusters was 200, the p-value was 3.735e-37.

Label	Mutation
Known	ABL1 ALK DNMT3A EGFR ERBB2 FGFR2 FGFR3 FLT3 GNAQ HRAS IDH2 JAK2 KIT MYC MYD88 NPM1 NRAS RB1 RUNX1 SDHB SMO
Predicted	ASXL1 BAP1 BCL6 CALR CCND1 CEBPA ETV6 FGFR1 H3F3A IKZF1 MET MYCN NF2 NOTCH1 NTRK1 PAX5 PDGFRA PPM1D RET RHOA ROS1 SMARCB1 TET2 WT1
Candidate	BCL2 CISH CRLF2 DUSP22 ERG EWSR1 FHIT MAML2 MYBL1 MYCL PDGFRB PLAG1 PRKACA SPINK1 SS18 TERT TFE3 TP63

The number of clusters was 300, the p-value was 1.806e-39.

Label	Mutation
Known	ALK CSF3R DNMT3A EGFR ERBB2 FGFR2 FGFR3 FLT3 GNA11 GNAQ HRAS IDH2 JAK2 KIT MAP2K1 MYD88 NPM1 NRAS SMO
Predicted	ARAF ASXL1 CALR CEBPA CSF1R FGFR1 H3F3A MET NOTCH1 PDGFRA RET RHOA TET2
Candidate	RIT1

The number of clusters was 500, the p-value was 2.894e-19.

Label	Mutation
Known	BRCA1 BRCA2 MLH1 MSH2 MSH6 MUTYH PMS2 RB1 SDHB
Predicted	BAP1 CHEK2 PALB2
Candidate	FHIT KLLN POLD1

6. Spectral Clustering

The number of clusters was 50, the p-value was 1.274e-09.

Label	Mutation
Known	ABL1 AKT1 ALK CDK4 ERBB2 FLT3<br>JAK2 KIT MAP2K1 MAP2K2 MTOR
Predicted	AKT2 ARAF BTK CDK6 FGFR1 INPP4B<br>IRS2 JAK1 JAK3 KDR LDHA MAP4K3<br>MARK2 PDGFRA PIK3CB PIK3R2 PIK3R3 PPM1D<br>RAC1 RAF1 RHEB RHOA ROS1 SMAD2<br>SMAD3 SOS1 SOS2 SRC STAT3 STAT5B<br>STK4 SYK TSC1 TSC2 WNK1
Candidate	AAK1 ABI1 ABI2 AKT1S1 AKT3 ARPP19<br>ASAH1 AURKA AURKB AXL BAD BCAR1<br>BIN2 BMX CDC25A CDC25B CDC25C CDC37<br>CDC42 CDC7 CDK1 CDK16 CDK19 CDK2<br>CDK3 CDK5 CDK7 CDK8 CDK9 CHEK1<br>CHKA CHMP4C CLK1 CRK CRKL CRLF2<br>CSK DAPK2 DAPK3 DCBLD2 DUSP2 DUSP3<br>DUSP4 DUSP5 DUSP6 DVL2 DYRK1A DYRK1B<br>DYRK2 DYRK3 ECT2 EIF2A EIF2AK2 EIF4EBP1<br>FRS2 FYN GAB1 GRB2 GRK6 GRK7<br>GSK3B GSKIP HCK HDAC6 HIPK1 HIPK2<br>HIPK3 IGF1R ILK IPMK IPPK IRAK4<br>ITK JUN KSR1 LATS1 LATS2 LCK<br>LIMK1 LIMK2 LMTK3 LYN MAP3K8 MAP4K4<br>MAPK1 MAPK11 MAPK13 MAPK14 MAPK3 MAPK7<br>MAPKAPK2 MAPKAPK3 MARCKS MARK3 MARK4 MASTL<br>MELK MLKL MOB1B MST1 NEK2 NEK6<br>NEK7 NUAK1 NUAK2 NUMB OR5P2 OSR1<br>PAGE4 PAK1 PAK2 PAK3 PAK4 PAK6<br>PASK PDE3A PDE3B PDE5A PDGFRB PDK1<br>PDK2 PDPK1 PFKFB3 PFKFB4 PHLPP1 PHLPP2<br>PI3 PIK3IP1 PIM1 PIM2 PIM3 PIP5K1C<br>PKD1 PKD2 PKM PKN1 PLD1 PLD2<br>PLK1 PLK2 PLK3 PLK4 PNKP POMK<br>PPM1A PPM1B PPP1R12A PRKACA PRKX PTK6<br>PTPN1 RALA RICTOR RIN1 RIPK1 RIPK2<br>RIPK3 RNF41 ROCK1 ROCK2 ROR2 RPS6<br>RPS6KB1 SAV1 SGK1 SGK2 SGK3 SIK1<br>SIK2 SMAD1 SMAD5 SMG1 SP4 SPHK1<br>SRMS SRPK1 SRPK2 STAT5A STK38 STRAP<br>TAB1 TAB3 TANK TBC1D1 TBC1D4 TBK1<br>TNNI3K TPK1 TPX2 TRAF4 TSSK2 TTBK2<br>TTK TYK2 ULK1 ULK2 VASP VAV1<br>VRK1 VRK2 WEE1 YAP1

The number of clusters was 100, the p-value was 4.696e-13.

Label	Mutation
Known	CSF3R DICER1 FGFR3 GNAS MYD88 NF1 NPM1 PTPN11 RB1 SDHB STK11 VHL
Predicted	ASXL1 BAP1 BMPR2 CALR CDC73 CEBPA EIF2AK3 ETNK1 ETV6 EXT1 EXT2 FBXW7 FLCN FMR1 GJB2 IKZF1 KCNJ5 MED12 MEN1 NF2 NSD1 PDGFRA PRKAR1A PTCH1 RASA1 RET SDHC SDHD SETBP1 SMARCB1 SMARCE1 TMEM127 TSC1 TSC2
Candidate	ABCA4 ABCD1 ACTN4 AGXT AIP AIRE AMELY ANO10 ANO5 ARMC5 ARX ATP6V1B1 AVPR2 BSCL2 C12orf65 C19orf12 CACNA1A CASR CDKL5 CHCHD10 CHD7 CLCN1 CLCN5 CLCNKB COL4A5 CTNS CTSC CYBB CYP21A2 DARS2 DKC1 DPY19L2 EFNB1 ELANE EWSR1 EXOSC3 EYA1 FBN1 FERMT1 FOXG1 FRMD7 GCK GHRHR GNRHR GPR101 GYG1 HCCS HFE HNF1B IL36RN KAL1 KCNJ11 KHDC3L KRIT1 LCA5 LMNA MECP2 MEFV MNX1 MPV17 MTM1 NLRP7 NOTCH3 NPHP1 NPHS1 NPHS2 NR0B1 NR5A1 OCRL PANK2 PDHA1 PHKG2 PIGN PKD1 PKD2 PLA2G6 POLG PORCN PROP1 PRRT2 RAI1 RIT1 SCN1A SF3B4 SFTPC SGCE SH3BP2 SHOX SIL1 SIM1 SLC12A3 SLC25A13 SLC26A4 SLC29A3 SLC2A1 SLC34A3 SLC3A1 SLC7A9 SLC9A6 SPAST SPINK1 STX16 STXBP1 SURF1 TBC1D24 TCOF1 THAP1 THRA THRB TTC19 UNC13D WDR45 WFS1 WISP3 YWHAE

The number of clusters was 200, the p-value was 4.575e-38.

Label	Mutation
Known	ABL1 ALK CSF3R EGFR ERBB2 FGFR2 FGFR3 FLT3 GNA11 GNAQ HRAS IDH2 JAK2 KIT MAP2K1 MYC MYD88 NPM1 NRAS PTPN11 SMO
Predicted	ARAF ASXL1 BTK CALR CCND1 CD79B CSF1R FGFR1 FGFR4 H3F3A JAK1 JAK3 MET MYCN NOTCH1 NTRK1 PDGFRA PPM1D RET ROS1 SETBP1 SMARCB1 SRC STAT3
Candidate	AXL BCL2 CRKL CRLF2 EPOR IGF1R MYCL PDGFB PDGFRB PRKACA REL RIT1 TERT

The number of clusters was 300, the p-value was 1.820e-29.

Label	Mutation
Known	CSF3R DNMT3A FLT3 GNA11 GNAQ GNAS HRAS IDH2 JAK2 KIT MAP2K1 MYD88 NPM1 NRAS PTPN11 STK11
Predicted	ARAF ASXL1 CALR CD79B CEBPA EIF1AX H3F3A KCNJ5 MPL NOTCH1 PDGFRA POLE SDHC SDHD SETBP1 TET2 TMEM127
Candidate	GNA14 GNB1 KHDC3L PDGFRB RBMS2 RIT1 SHOC2 SPRED1 ZNF764

The number of clusters was 500, The p-value was 9.575e-18.

Label	Mutation
Known	AKT1 CSF3R GNA11 GNAQ HRAS MAP2K1 MYD88 PTPN11 STK11
Predicted	ACVR1 ARAF CD79B EIF1AX H3F3A SETBP1
Candidate	PDGFRB PRKACA RIT1 SHOC2 SPRED1

References

[1] Gonzalez-Perez, A., Perez-Llamas, C., Deu-Pons, J., Tamborero, D., Schroeder, M.P., Jene-Sanz, A., Santos, A., Lopez-Bigas, N.: IntOGen-mutations identifies cancer drivers across tumor types. Nature methods **10**(11), 1081–1082 (2013)

[2] Pedregosa, F., Varoquaux, G., Gramfort, A., Michel, V., Thirion, B., Grisel, O., Blondel, M., Prettenhofer, P., Weiss, R., Dubourg, V., Vanderplas, J., Passos, A., Cournapeau, D., Brucher, M., Perrot, M., Duchesnay, E.: Scikit-learn: Machine learning in Python. Journal of Machine Learning Research **12**, 2825–2830 (2011)
